# Circulating miRNAs and Vascular Injury Markers Associate with Cardiovascular Function in Older Patients Reaching End-Stage Kidney Disease

**DOI:** 10.3390/ncrna8010002

**Published:** 2022-01-10

**Authors:** Qiao Zhao, Sabine J. L. Nooren, Laurien E. Zijlstra, Jos J. M. Westenberg, Lucia J. M. Kroft, J. Wouter Jukema, Noeleen C. Berkhout-Byrne, Ton J. Rabelink, Anton Jan van Zonneveld, Marjolijn van Buren, Simon P. Mooijaart, Roel Bijkerk

**Affiliations:** 1 Department of Internal Medicine (Nephrology), Leiden University Medical Center, Albinusdreef 2, 2333 ZA Leiden, The Netherlands; q.zhao@lumc.nl (Q.Z.); sabine.nooren@gmail.com (S.J.L.N.); n.c.berkhout-byrne@lumc.nl (N.C.B.-B.); A.J.Rabelink@lumc.nl (T.J.R.); A.J.van_Zonneveld@lumc.nl (A.J.v.Z.); m.van_buren@lumc.nl (M.v.B.); 2 Einthoven Laboratory for Vascular and Regenerative Medicine, Leiden University Medical Center, Albinusdreef 2, 2333 ZA Leiden, The Netherlands; 3 Department of Cardiology, Leiden University Medical Center, Albinusdreef 2, 2333 ZA Leiden, The Netherlands; l.e.zijlstra@lumc.nl (L.E.Z.); J.W.Jukema@lumc.nl (J.W.J.); 4 Department of Radiology, Leiden University Medical Center, Albinusdreef 2, 2333 ZA Leiden, The Netherlands; j.j.m.westenberg@lumc.nl (J.J.M.W.); l.j.m.kroft@lumc.nl (L.J.M.K.); 5 Netherlands Heart Institute, Moreelsepark 1, 3511 EP Utrecht, The Netherlands; 6 Department of Nephrology, HAGA Hospital, 2545 AA The Hague, The Netherlands; 7 Department of Gerontology and Geriatrics, Leiden University Medical Center, 2300 RC Leiden, The Netherlands; S.P.Mooijaart@lumc.nl

**Keywords:** end-stage kidney disease (ESKD), cardiovascular disease (CVD), miRNAs, angiopoietin-2, pulse wave velocity (PWV)

## Abstract

The prevalence of end-stage kidney disease (ESKD) is rapidly increasing and mostly occurring in patients aged 65 years or older. The main cause of death in these patients is cardiovascular disease (CVD). Novel markers of vascular integrity may thus be of clinical value for identifying patients at high risk for CVD. Here we associated the levels of selected circulating angiogenic miRNAs, angiopoietin-2 (Ang-2) and asymmetric dimethylarginine (ADMA) with cardiovascular structure and function (as determined by cardiovascular MRI) in 67 older patients reaching ESKD that were included from ‘The Cognitive decline in Older Patients with End stage renal disease’ (COPE) prospective, multicentered cohort study. We first determined the association between the vascular injury markers and specific heart conditions and observed that ESKD patients with coronary heart disease have significantly higher levels of circulating ADMA and miR-27a. Moreover, circulating levels of miR-27a were higher in patients with atrial fibrillation. In addition, the circulating levels of the vascular injury markers were associated with measures of cardiovascular structure and function obtained from cardiovascular MRI: pulse wave velocity (PWV), ejection fraction (EF) and cardiac index (CI). We found Ang-2 and miR-27a to be strongly correlated to the PWV, while Ang-2 also associated with ejection fraction. Finally, we observed that in contrast to miR-27a, Ang-2 was not associated with a vascular cause of the primary kidney disease, suggesting Ang-2 may be an ESKD-specific marker of vascular injury. Taken together, among older patients with ESKD, aberrant levels of vascular injury markers (miR-27a, Ang-2 and ADMA) associated with impaired cardiovascular function. These markers may serve to identify individuals at higher risk of CVD, as well as give insight into the underlying (vascular) pathophysiology.

## 1. Introduction

Chronic kidney disease (CKD) has a worldwide prevalence of about 10%, which is rapidly increasing [1,2,3]. Eventually, these patients may develop end-stage kidney disease (ESKD) and become dependent on kidney replacement therapy (transplantation or dialysis). Currently, half of all new ESKD patients are aged 65 years or older [4,5]. These older patients with ESKD have an increased risk for adverse health events, such as cognitive impairment [6,7] and cardiovascular disease (CVD) [8,9,10,11]. The probability for an older patient with CKD to develop CVD is significantly greater than that of the normal population [9,12]. The risks for older patients with ESKD is even higher [13], where premature CVD is the main cause of death in these patients [8,11,14,15]. The high incidence of CVD in ESKD relates to traditional CVD-risk factors such as hypertension and diabetes (often present in CKD patients), but also CKD-specific alterations that may cause (micro)vascular dysfunction: increases in circulating levels of waste products such as uremic toxins (e.g., asymmetric dimethylarginine (ADMA), altered renal endocrine factors (renin-angiotensin-aldosterone-system, vitamin D, erythropoietin), anemia, serum calcium and phosphate levels [16,17,18]. These nephrogenic factors predispose these patients to eventually develop abnormal cardiovascular structure or function [14,15,16,17,18,19], as defined by arterial stiffness (pulse wave velocity (PWV)), systolic heart function (cardiac index (CI) and the ejection fraction (EF)), and eventually heart failure [20,21]. However, the exact underlying pathophysiological mechanisms behind the highly frequent impaired cardiovascular structure and function in ESKD patients remain unclear.

**Figure 1:**
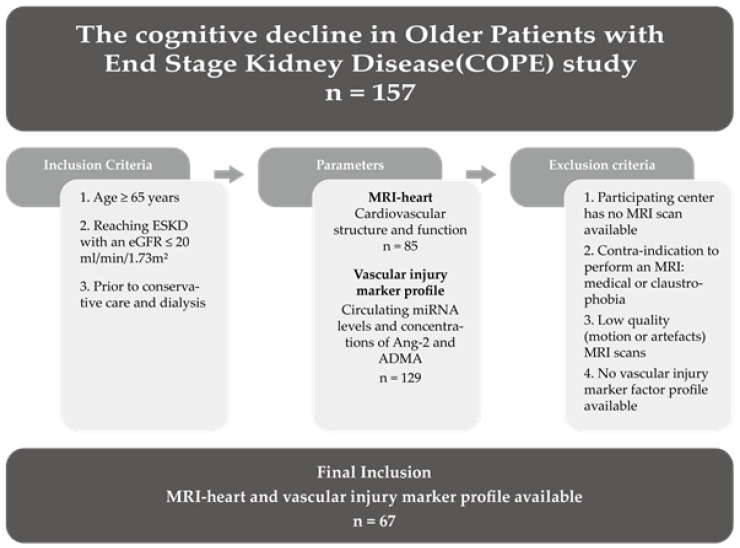
Flow chart of study population selected from the COPE study. The COPE study contained 157 older patients with end-stage kidney disease (ESKD), from which multiple parameters were measured. This study only included the patients with available MRI scans of the heart and with a vascular injury marker profile. Other patients were excluded, which resulted in a study population of 67 patients.

Vascular injury related factors such as angiopoietin-2 (Ang-2) and ADMA, together with (circulating) angiogenic miRNAs, play a major role in mediating vascular injury or maintaining microvascular integrity [22,23,24,25,26]. These circulating miRNAs can bind to messenger RNAs in target cells to inhibit their translation and thereby directly regulate important pathways in maintaining microvascular integrity [24,27]. As such, assessing their circulating levels in disease may provide insight into the underlying vascular pathophysiology, as well as provide potential markers for development of (micro)vascular injury and CVD [26,28,29,30,31]. For instance, circulating miRNAs can serve as biomarkers for myocardial infarction (miR-208) [32] and heart failure (miR-423) [33]. MiRNAs to be assessed in this study (miR-27a, miR-29a, miR-126, miR-132, miR-137, miR-192, miR-223 and miR-326) were selected based on their previously determined relation to vascular injury and cognitive dysfunction [31]. For instance, it has been shown that angiogenic miR-126 contributes to the maintenance of vascular integrity by modulating the mobilization of hematopoietic progenitor cells, thereby initiating vascular regeneration [22]. Heart failure associated miR-132 and miR-192 were described to impact capillary density through regulating angiogenic signaling pathways [23,34,35]. MiR-223, miR-326 and miR-27a were reported to be, amongst others, associated with diabetic vascular complications [30], while miR-29 can restore normal endothelial cell function in cardiometabolic disorders [36]. In the present study, we aimed to investigate whether these selected circulating angiogenic miRNAs and the vascular injury markers Ang-2 and ADMA are also directly correlated with cardiovascular structure and function in older patients with ESKD (study overview in Figure 1). For that purpose, we accessed the COPE study [37], a unique cohort of patients ≥65 years of age reaching ESKD [(eGFR) ≤ 20 mL/min/1.73 m^2^] before receiving kidney replacement therapy in which comprehensive CVD testing was performed using cardiovascular MRI. These data allow us to study pathophysiological mechanisms of CVD in older patients reaching ESKD.

## 2. Materials and Methods

### 2.1. Patient Cohort

Patient data was gathered from ‘The Cognitive decline in Older Patients with End stage renal disease’ (COPE) prospective, multicentered cohort study. The design and rationale behind the COPE study with all in- and exclusion criteria has been published previously [37]. In brief, older patients with an age above 65, suffering from chronic kidney disease stage 4 or 5 (eGFR ≤ 20 mL/min/1.73 m^2^) and prior to any conservative care or dialysis, were included. To study the association between circulating miRNAs and alterations in cardiovascular structure and function, blood samples and cardiovascular magnetic resonance imaging (MRI) scans were taken. Patients without a cardiovascular MRI, either due to the lack of an available MRI at the participating center, contra-indications to perform an MRI, or an unusable MRI due to motion or artefacts, were excluded from this study (Figure 1). All included patients signed a written informed consent and the medical ethics committees (METC) approved the study protocol of all the four participating centers (Leiden University Medical Center [Leiden, LUMC], HAGA hospital [the Hague], HAGA hospital [Zoetermeer, dialysis center] and Reinier de Graaf Groups [Delft]).

### 2.2. Renal Care

All participating centers used either the Chronic Kidney Disease Epidemiology Collaboration (CKD-epi) [38] or the Modification of Diet in Renal Disease (MDRD) [39] to estimate the patient’s estimated glomerular filtration rate (eGFR). Based on the ERA-EDTA primary renal diagnosis code, patients were divided by the cause of their kidney disease, either non-vascular or vascular (mostly diabetes or hypertension).

### 2.3. Magnetic Resonance Imaging (MRI)

All cardiovascular MRI scans were made on a 3T Philips Achieva MRI scanner (Philips, Best, The Netherlands) with an eight-channel receive coil.

### 2.4. Cardiovascular Structure and Function

Phase-contrast MRI scans, which visualize moving fluid, were used to determine the pulse wave velocity (PWV), which was required to measure the aortic stiffness of the patients [37]. Moreover, turbo field echo (TFE) multi-slice multi-phase cine-imaging of the left ventricle was made to measure the ejection fraction (EF) and cardiac index (CI), the two parameters of the cardiac systolic function. The EF is the percentage of blood leaving the left ventricle each time the heart contracts (stroke volume (SV)/end-diastolic volume (EDSV) * 100%). The CI is expressed in L/min/m^2^ and determined by correcting the cardiac output (CO, stroke volume (SV) * heart rate (HR)) for the body surface area (BSA) with the Du Bois formula [40]. For only one patient, the CI could not be determined, due to a low-quality MRI scan. PWV and EF were available for all 67 patients.

### 2.5. Angiopoietin-2 II (Ang-2) and Asymmetric Dimethylarginine (ADMA)

An ELISA (R&D Systems, Minneapolis, MN, USA) was performed to measure the serum concentrations of the vascular injury markers Ang-2 and ADMA.

### 2.6. Circulating Angiogenic miRNAs

Patients EDTA-anti-coagulated blood was centrifugated for 5 min at 3000 rcf to harvest plasma from the 67 patients for the miRNA analysis. After sample collection, the plasma was stored at −80 °C. MicroRNA measurements were performed by Exiqon (Vaedbek, Denmark) with the total amount of RNA isolated from 200 µL via RT-qPCR using corresponding ‘SYBR Green based miRCURY LNA PCR assays’. Then, 2 μL RNA was reverse transcribed in 10 μL reactions using the miRCURY LNA Universal RT microRNA PCR, Polyadenylation and cDNA synthesis kit (Exiqon; catalog #203301). cDNA was diluted 50× and assayed in 10 μL PCR reactions according to the protocol for miRCURY LNA™ Universal RT microRNA PCR; each microRNA was assayed once by qPCR on the microRNA Ready-to-Use PCR, Custom Pick and Mix panel using ExiLENT SYBR Green master mix. Negative controls excluding template from the reverse transcription reaction was performed and profiled like the samples. The amplification was performed in a LightCycler^®^ 480 Real-Time PCR System (Roche) in 384 well plates. Selection of the miRNAs to be tested (miR-27a, miR-29a, miR-126, miR-132, miR-192, miR-223 and miR-326) was based on a previous study [31], which related these miRNAs to an impaired cognitive function and vascular injury. For normalization of the data, we have applied the median of the assays detected in all samples as this was found to be the most stable normalizer [31].

### 2.7. Statistical Analysis

All data analyses were performed with IBM SPSS Statistics version 25. The cut-off values to divide the cardiovascular parameters PWV, EF and CI into two dichotomized groups are based on current guidelines [41,42]. The presence of elevated aortic stiffness was determined by a PWV > 10 m/s and a bad EF and CI characterized by <50% and ≤2.2 L/min/m^2^, respectively. Categorical data are given in numbers with percentages and all continuous data are presented as mean ± standard deviation (SD) or median ± interquartile range (IQR). Independent-sample T tests were used to assess the baseline differences between the cardiovascular parameters. Correlation models were used to assess the correlation between the circulating miRNA levels and alterations in cardiac structure and function. Through testing it was found that gender and age (likely due to the small age range in our cohort) did not represent significant confounders, and as such no adjustments were performed for these parameters. Given the exploratory nature of our studies, no correction for multiple testing was performed and statistical significance was considered with *p*-values < 0.05.

## 3. Results

### 3.1. Patient Cohort Description

67 patients of the COPE study had a cardiovascular MRI scan and microRNA profile available and were included in this study (Figure 1).

Table 1 shows the baseline clinical characteristics of this study population. The mean (±SD) age of this population was 75.1 years (±6.6 years) and 44 (65.7%) participants were male. At baseline, the mean (±SD) eGFR was 16.0 mL/min/1.73 m^2^ (±4.0 mL/min/1.73 m^2^) and the primary kidney disease had a vascular cause, mainly diabetes or hypertension, in 41 (61.2%) participants. The median [IQR] of the cardiovascular function parameters was 9.8 m/s [8.0–13.7] for the pulse wave velocity (PWV), 61% [51–66] for the ejection fraction (EF) and 2.5 L/min/m^2^ [2.1–3.0] for the cardiac index (CI). A history of cardiovascular comorbidity was present in 40 patients (59.7%; Table 1).

### 3.2. Associations between Vascular Injury Markers and Cardiovascular Disease

We first assessed whether a vascular cause of the primary kidney disease was associated with different levels of vascular injury markers, namely circulating angiogenic miRNAs, angiopoietin II (Ang-2) and asymmetric dimethylarginine (ADMA). As illustrated in Table 2, no significant associations were observed, although a trend towards lower miR-27a levels was found in the group with a vascular cause of CKD (*p* = 0.074). Next, to investigate whether the vascular injury markers associated with a higher risk of cardiovascular disease, we compared the concentrations and levels of the vascular injury markers in older patients with ESKD with or without a specific cardiovascular condition: heart failure (HF), coronary heart disease (CHD), left ventricular hypertrophy (LVH) or atrial fibrillation (AF) (Table 2). No significant differences were seen in the circulating miRNA levels of miRNA-126, -132, -192, -29a and -326 between the patients with or without any of these cardiovascular conditions.

In contrast, the concentration of the vascular injury marker ADMA was significantly higher in older patients with ESKD and CHD (*p* = 0.027). Moreover, the circulating angiogenic miR-27a was significantly higher in older patients with ESKD and either CHD (*p* = 0.044) or AF (*p* = 0.016) compared to those patients with ESKD without this condition. In addition, we found trends for associations of higher Ang-2 with presence of HF (*p* = 0.081), miR-223 with CHD (*p* = 0.058), and miR-326 with AF (*p* = 0.089).

### 3.3. Associations between Vascular Injury Markers and Cardiovascular Function

Table 3 shows the correlations and associations between the cardiovascular function parameters and the vascular injury markers. To assess associations, we first used cut-off values to divide the cardiovascular parameters into two dichotomized groups (based on clinically relevant cut-off values) and tested whether the levels of the vascular injury markers were significantly different between the two groups. Second, we assessed the (continuous) correlation between injury marker and cardiovascular function parameters.

For the dichotomized groups, we observed that for a higher PWV (>10 m/s), which indicates a stiffer aorta, the circulating levels of angiogenic miR-27a were significantly lower compared to the group with a PWV <10 m/s (*p* = 0.012), while circulating levels of Ang-2 showed a trend towards higher levels in the high PWV group (*p* = 0.083). No statistically significant differences were found in the dichotomized CI groups and EF groups. However, we found a trend of higher circulating miR-326 levels in the group with EF < 50% (*p* = 0.061).

For the continuous correlation assessment, two statistically significant correlations between the PWV and the vascular injury markers were found, namely with Ang-2 and miR-27a. A higher PWV was strongly correlated with higher serum Ang-2 concentrations (r = 0.45, *p* = 0.000) and with lower circulating levels of miR-27a (r = −0.389, *p* = 0.001). Furthermore, the circulating levels of Ang-2 were found to be negatively correlated with ejection fraction (r = −0.250, *p* = 0.035). In addition, we found a trend towards an inverse correlation of miR-326 with EF (*p* = 0.079).

**Figure 2:**
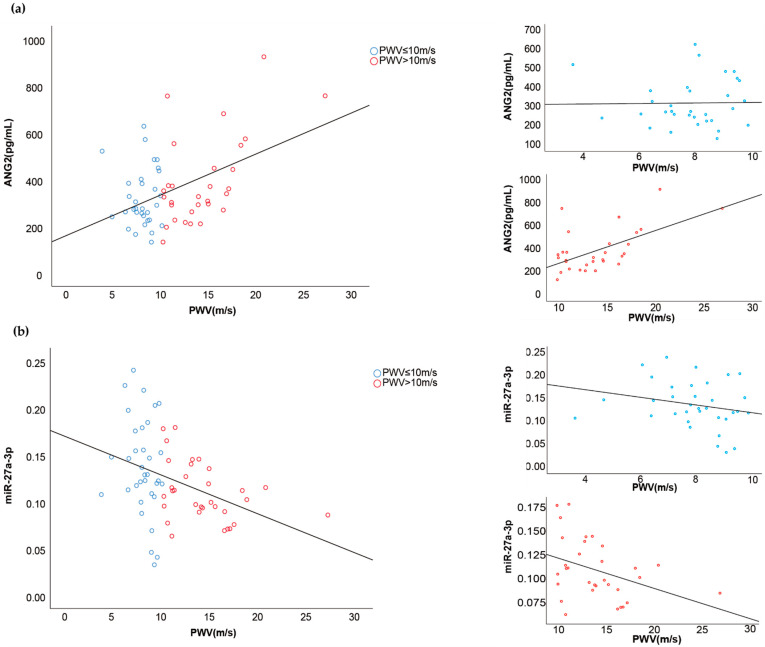

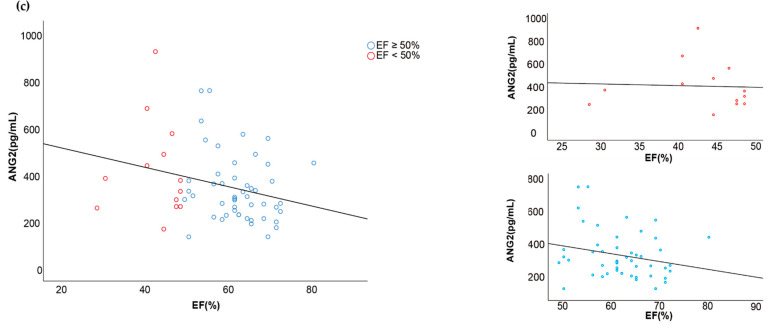
Correlations between PWV and the vascular injury markers Ang-2 and miR-27a. Blue dots represent better cardiovascular condition patients whose PWV ≤ 10 m/s and red dots represent worse cardiac condition patients whose PWV > 10 m/s. (**a**) PWV was significantly correlated with serum Ang-2 concentrations (R = 0.45, *p* = 0.000) and the correlation between PWV and Ang-2 remained significant (R = 0.583, *p* = 0.001) when PWV > 10 m/s, while not existed (R = 0.016, *p* = 0.913) when PWV ≤ 10 m/s. (**b**) Higher PWV was significantly correlated with miR-27 (R = −0.389, *p* = 0.001) and correlation remained significant (R = −0.379, *p* = 0.033 respectively) within the group with a PWV > 10 m/s. There is no significant correlation between PWV and miR-27 (R = −0.232, *p* = 0.102) when PWV < 10 m/s. (**c**) EF was significantly correlated with Ang-2 (R = −0.250, *p* = 0.035), but the correlation was not significant either in the group with EF ≥ 50% (R = −0.229, *p* = 0.113, respectively) or in the group with EF < 50% (R = −0.046, *p* = 0.880).

Next, we aimed to assess whether these statistically significant correlations were different in the dichotomized groups (i.e., PWV either higher or lower than 10 m/s; EF either higher or lower than 50%). As such, when the miR-27a and Ang-2 correlations (with PWV) were tested in the group with a relatively good PWV (≤10 m/s) or bad PWV (>10 m/s) separately, the discovered correlation between PWV and Ang-2 and correlation between PWV and miR-27a remained significant (r = 0.583, *p* = 0.001; r = −0.379, *p* = 0.033, respectively) within the group with a PWV > 10 m/s (Figure 2a,b). Interestingly, when testing the correlations between the two mentioned vascular injury markers and PWV within the group with a PWV ≤ 10 m/s, the correlation between PWV and Ang-2 or miR-27a was not statistically significant anymore (r = 0.016, *p* = 0.931; r = −0.232, *p* = 0.187, respectively). The correlation between Ang-2 and EF was no longer statistically significant when analyzed in the EF < or > 50% groups separately (Figure 2c).

These findings prompted us to test if the other markers correlated with specific parameters in specific dichotomized groups. Indeed, we observed miR-132 to be positively correlated with PWV within the group of PWV > 10 m/s (R = 0.371, *p* = 0.043), while miR-223 negatively correlated with CI within the group of CI ≤ 2.2 L/min/m^2^ (R = −0.440, *p* = 0.046) (Supplementary Figure S1). No other significant correlations or associations were observed between these vascular injury markers and the CI, nor with the EF (Supplementary Table S1).

## 4. Discussion

This study revealed that, in older patients reaching ESKD, vascular injury markers are associated with markers of cardiovascular structure and function. Specifically, the main findings were (1) higher levels of circulating angiogenic miR-27a associate with presence of AF or CHD, (2) increased concentration of ADMA in patients with CHD, (3) a positive correlation between a higher PWV and Ang-2 and a negative correlation between a higher PWV and circulating miR-27a, and (4) a negative correlation between Ang-2 levels and lower EF.

When we assessed the correlations between PWV and Ang-2/miR-27a within the high (>10 m/s) and low (≤10 m/s) groups separately, these correlations were even stronger (as illustrated by a higher r) within the high PWV group, suggesting the discovered correlations are mostly based on the group with a high PWV (>10 m/s). This could possibly be explained by the Windkessel effect of the arteries that is compromised in patients with arterial stiffness. The Windkessel effect of normal elastic arteries, such as the aorta, decreases the pulsatility of the blood pump out of the heart, converts it into a more constant outflow and thereby prevents damage to the microvasculature [21]. As this effect is compromised in patients with a PWV > 10 m/s, their microvasculature has to cope with a higher pulsatility, which could initiate the local endothelial cells to start producing more Ang-2 [43,44]. Indeed, Ang-2 was experimentally demonstrated to be involved in arterial stiffness [45]. Similarly, low miR-27a levels were demonstrated to be causally involved in vascular remodeling [46] and vascular calcification [47] via regulating vascular smooth muscle cell phenotype, potentially explaining its link we observed here with PWV. Interestingly, miR-27a has been shown to inhibit the production of angiotensin-converting enzyme (ACE) [48,49], which converts angiotensin-1 into angiotensin-2, while angiotensin-2 can stimulate the expression of angiopoietin-2 (Ang-2) [50], suggesting a possible direct link between Ang-2 and circulating miR-27a levels. Taken together, a high concentration of Ang-2, or low miR-27a levels, could potentially serve as a biomarker for aortic stiffness and may reflect underlying pathophysiology. In addition, it would be interesting to dissect whether these altered Ang-2 and miR-27a levels are ESKD-specific or related to (other) systemic vascular injury. In that sense, the fact that miR-27a levels appear lower in patients with a primary kidney disease with a vascular cause, while Ang-2 levels are not dependent on the cause of the primary kidney disease, suggests Ang-2 might be an ESKD-induced factor that may drive further CVD. 

We previously studied in the same cohort the association between cardiovascular structure and function, vascular injury markers and measures of cognitive function [7,31]. We observed strong positive correlations of Ang-2 [31] and PWV [7] with cognitive function in the domains of executive function and psychomotor speed, as well as a negative correlation between miR-27a and executive function [31]. Together with the observed Ang-2 and miR-27a association with PWV in the present study, these findings suggest an interesting link for the presumed shared pathophysiology in microvascular damage in the heart-kidney-brain axis. Similarly, we here found miR-223 to correlate to cardiac index (only in the low cardiac index group, Supplementary Figure S1), while we previously demonstrated that both miR-223 and cardiac index associated with memory function [7,31]. 

We also observed that miR-27a levels were higher in older patients with ESKD and AF or CHD. This appears in line with previous findings that miR-27a could potentially be a biomarker for atherosclerosis as its levels correlated with the progression of atherosclerosis [51,52]. Moreover, several studies found that an increased arterial stiffness was significantly correlated with the presence of AF (although we could not confirm this in our study, data not shown) [53,54]. However, we observed a negative correlation between miR-27a levels and arterial stiffness (PWV), thus contradicting a direct link in our studies between miR-27a, PWV and AF. Dedicated studies are therefore necessary to clarify the exact link between miR-27a and the development of different cardiovascular diseases. It should also be noted that our analysis of vascular injury markers in relation to these specific heart conditions should be carefully interpreted as these conditions involve small groups (e.g., the number of ESKD patients with CHD is only 15). Indeed, a limitation of our study is the limited group sizes.

Taken together, this study shows the potential of circulating angiogenic miRNAs, Ang-2 and ADMA to serve as biomarkers for cardiovascular structure and function in older patients reaching ESKD. Interestingly, Ang-2 levels are not dependent on the (vascular) cause of the primary kidney disease, suggesting that Ang-2 might be an ESKD-induced factor that may drive further CVD. Finally, further research into the found correlations could give more insight in the role of the vascular injury markers in impaired cardiovascular function.

## Figures and Tables

**Table 1:** Baseline characteristics of the study population (n = 67).

Male gender, n (%)	44 (65.7)
Age, years; mean ± SD	75.1 ± 6.6
Body mass index (BMI); mean ± SD	27.9 ± 3.83
Race, Caucasian, n (%)	58 (86.6)
Higher educational level ^1^, n (%)	25 (37.4)
Primary kidney disease, n (%)	
Non-vascular cause	25 (37.3)
Vascular cause	41 (61.2)
Comorbidity, n (%)	
Diabetes mellitus	28 (41.8)
Peripheral vascular disease	10 (14.9)
Cerebral vascular accident	17 (25.4)
Heart failure	4 (6.0)
Coronary heart disease	15 (22.4)
Left ventricle hypertrophy	8 (11.9)
Atrial fibrillation	14 (20.9)
Alcohol consumption	39 (58.2)
History of smoking	39 (72.2)
Medication use, n (%)	
Polypharmacy (the use of ≥5 medications)	62 (92.5)
Antihypertensive medication	62 (92.5)
Diuretics	38 (56.7)
Cholesterol-lowering	49 (73.1)
Anti-coagulants	50 (74.6)
Objective measures, mean ± SD	
Blood pressure (mmHg)	
Systolic	155.7 ± 21.7
Diastolic	81.6 ± 11.3
eGFR (mL/min/1.73 m^2^)	16.0 ± 4.0
Urea (mmol/L)	21.3 ± 6.6
Albumin (mg/24 h) ^2^	803 ± 956
Troponin-T (µg/L)	0.071 ± 0.117
NT-proBNP (ng/L)	792 ± 1155
Cholesterol	4.5 ± 1.1
HDL (mmol/L)	1.22 ± 0.39
LDL-cholesterol (mmol/L)	2.49 ± 0.80
Cardiovascular function, measured by MRI, median [IQR]	
Pulse wave velocity (m/s)	
Ejection fraction (%)	9.8 [8.0–13.7]
Cardiac index (L/min/m^2^)	61 [51–66]
	2.5 [2.1–3.0]

^1^ Higher education level includes HAVO/WVO/HBO and university. ^2^ Missing values of 29 patients. Abbreviations: estimated glomerular filtration rate (eGFR); high-density-lipoprotein (HDL); interquartile range (IQR); low-density-lipoprotein (LDL); magnetic resonance imaging (MRI); N-terminal pro b-type natriuretic peptide (NT-proBNP); standard deviation (SD).

**Table 2:** Associations between vascular injury markers and cardiovascular conditions.

	**Non-Vascular Cause Primary Kidney Disease (n = 25)**		**Vascular Cause Primary** **Kidney Disease (n = 42)**		***p* Value**
	**mean**	**SD**	**mean**	**SD**	
ADMA	0.726	0.190	0.716	0.203	0.837
ANG2	321.502	145.797	366.568	166.078	0.287
miR-126	0.950	0.277	0.962	0.293	0.866
miR-132	0.010	0.006	0.012	0.008	0.285
miR-192	0.009	0.014	0.008	0.008	0.848
miR-223	3.867	1.859	3.764	1.077	0.802
miR-27a	0.134	0.036	0.115	0.046	0.074
miR-29a	0.027	0.036	0.027	0.031	0.981
miR-326	0.005	0.003	0.008	0.008	0.148
	**ESKD without HF (n = 63)**		**ESKD with HF (n = 4)**		***p* Value**
	**mean**	**SD**	**mean**	**SD**	
ADMA	0.712	0.195	0.824	0.198	0.273
ANG2	344.666	157.669	490.254	179.785	0.081
miR-126	0.952	0.286	0.995	0.292	0.772
miR-132	0.011	0.007	0.009	0.005	0.682
miR-192	0.008	0.011	0.009	0.006	0.901
miR-223	3.790	1.365	4.278	2.163	0.505
miR-27a	0.122	0.043	0.156	0.056	0.133
miR-29a	0.028	0.033	0.018	0.015	0.576
miR-326	0.006	0.006	0.012	0.015	0.532
	**ESKD without CHD (n = 52)**		**ESKD with CHD (n = 15)**		***p* Value**
	**mean**	**SD**	**mean**	**SD**	
ADMA	0.691	0.174	0.816	0.237	0.027 *
ANG2	350.111	161.698	367.596	166.662	0.725
miR-126	0.947	0.302	0.980	0.218	0.692
miR-132	0.012	0.008	0.008	0.004	0.086
miR-192	0.009	0.011	0.007	0.010	0.684
miR-223	3.644	1.412	4.424	1.246	0.058
miR-27a	0.118	0.043	0.145	0.045	0.044 *
miR-29a	0.030	0.035	0.017	0.014	0.178
miR-326	0.007	0.006	0.007	0.008	0.958
	**ESKD without LVH (n = 59)**		**ESKD with LVH (n = 8)**		***p* Value**
	**mean**	**SD**	**mean**	**SD**	
ADMA	0.728	0.200	0.651	0.155	0.299
ANG2	359.281	168.427	305.318	58.771	0.442
miR-126	0.935	0.273	1.098	0.340	0.128
miR-132	0.011	0.008	0.011	0.005	0.961
miR-192	0.007	0.006	0.016	0.025	0.331
miR-223	3.858	1.425	3.525	1.303	0.534
miR-27a	0.126	0.046	0.107	0.023	0.25
miR-29a	0.028	0.034	0.022	0.019	0.618
miR-326	0.007	0.007	0.004	0.002	0.27
	**ESKD without AF (n = 53)**		**ESKD with AF (n = 14)**		***p* Value**
	**mean**	**SD**	**mean**	**SD**	
ADMA	0.704	0.190	0.775	0.212	0.233
ANG2	329.794	125.840	437.253	235.965	0.122
miR-126	0.959	0.293	0.937	0.254	0.797
miR-132	0.011	0.008	0.010	0.005	0.624
miR-192	0.009	0.012	0.007	0.006	0.594
miR-223	3.681	1.265	4.339	1.807	0.121
miR-27a	0.117	0.040	0.149	0.053	0.016 *
miR-29a	0.025	0.028	0.035	0.046	0.321
miR-326	0.006	0.006	0.010	0.009	0.089 #

Independent-samples T tests were used for calculating *p*-values. Unit of ADMA is umol/L and unit of Ang-2 is pg/mL. The results were shown as mean ± SD. * Indicates statistically significant values (*p* < 0.05), # indicates trends (*p* < 0.10). ESKD = end stage kidney disease; HF = heart failure; CHD = coronary heart disease; LVH = left ventricular hypertrophy; AF = atrial fibrillation.

**Table 3:** Associations between vascular injury markers and cardiovascular function parameters.

	Better Cardiovascular Function	Worse Cardiovascular Function			
	Pulse Wave Velocity ≤ 10 m/s (n = 35)	Pulse Wave Velocity > 10 m/s (n = 32)	Correlation		*t*-Test
			*p* Value	R	*p* Value
ADMA		0.73 ± 0.20	0.787	0.034	0.547
Ang-2	319.6 ± 123.7	390.8 ± 189.4	0.000 *	0.451	0.083 #
miR-126	0.947 ± 0.324	0.963 ± 0.237	0.849	−0.013	0.818
miR-132	0.011 ± 0.008	0.011 ± 0.006	0.169	0.190	0.970
miR-192	0.009 ± 0.014	0.007 ± 0.006	0.999	0.000	0.561
miR-223	3.850 ± 1.442	3.784 ± 1.388	0.217	−0.149	0.849
miR-27a	0.137 ± 0.051	0.110 ± 0.031	0.001 *	−0.389	0.012 *
miR-29a	0.027 ± 0.032	0.027 ± 0.033	0.645	0.112	0.991
miR-326	0.008 ± 0.008	0.006 ± 0.004	0.798	0.006	0.241
	**Ejection Fraction ≥ 50% (n = 53)**	**Ejection Fraction < 50% (n = 14)**			
ADMA	0.72 ± 0.20	0.70 ± 0.16	0.530	0.076	0.721
Ang-2	337.3 ± 144.9	417.1 ± 208.1	0.035 *	−0.250	0.114
miR-126	0.944 ± 0.294	0.996 ± 0.245	0.463	−0.098	0.555
miR-132	0.011 ± 0.007	0.011 ± 0.008	0.857	−0.027	0.813
miR-192	0.008 ± 0.012	0.008 ± 0.005	0.941	−0.047	0.946
miR-223	3.766 ± 1.406	4.036 ± 1.442	0.431	−0.152	0.539
miR-27a	0.125 ± 0.043	0.120 ± 0.054	0.779	0.000	0.754
miR-29a	0.029 ± 0.035	0.024± 0.014	0.243	0.110	0.665
miR-326	0.006 ± 0.006	0.010 ± 0.009	0.079 #	−0.216	0.061 #
	**Cardiac Index > 2.2 L/min/m^2^ (n = 45)**	**Cardiac Index ≤ 2.2 L/min/m^2^ (n = 21)**			
ADMA	0.74 ± 0.22	0.68 ± 0.14	0.269	0.143	0.257
Ang-2	339.2 ± 166.7	379.8 ± 153.9	0.817	−0.046	0.364
miR-126	0.930 ± 0.264	1.018 ± 0.322	0.479	−0.091	0.246
miR-132	0.011 ± 0.007	0.011 ± 0.007	0.548	−0.073	0.802
miR-192	0.009 ± 0.012	0.007 ± 0.007	0.987	0.010	0.555
miR-223	3.750 ± 1.312	4.018 ± 1.621	0.960	0.024	0.476
miR-27a	0.125 ± 0.043	0.121 ± 0.049	0.972	0.019	0.751
miR-29a	0.026 ± 0.030	0.030 ± 0.039	0.940	0.027	0.658
miR-326	0.007 ± 0.008	0.006 ± 0.004	0.931	−0.009	0.301

Bivariate correlation models were used for the correlation *p*-values. To compare the vascular injury marker concentrations/circulating levels between the low versus high pulse wave velocity (PWV), ejection fraction (EF) and cardiac index (CI) groups, independent-samples T tests were used for the *p*-values. Unit of ADMA is umol/L and unit of Ang-2 is pg/mL. * Indicates statistically significant values (*p* < 0.05), # indicates trends (*p* < 0.10).

## Data Availability

The data presented in this study are available on request from the corresponding author.

## Supplementary Materials

The following supporting information can be downloaded at: https://www.mdpi.com/article/10.3390/ncrna8010002/s1, Figure S1: Blue dots represent better cardiovascular condition patients whose PWV ≤ 10 m/s or CI > 2.2 L/min/m^2^, and red dots represent worse cardiac condition patients whose PWV > 10 m/s or CI ≤ 2.2 L/min/m^2^; Table S1: Correlations between vascular injury markers and cardiovascular function parameters within dichotomized groups.
