# Supplementary material for: Circulating miRNAs and Vascular Injury Markers Associate with Cardiovascular Function in Older Patients Reaching End-Stage Kidney Disease
Source: Noncoding RNA. 2022 Jan 10;8(1):2. doi: 10.3390/ncrna8010002 (PMC8788543; doi:10.3390/ncrna8010002)
Supplement: Supplementary file 1 [file ncrna-08-00002-s001.zip › ncrna-1530108-supplementary.pdf]

Supplementary Figure S1

(a)

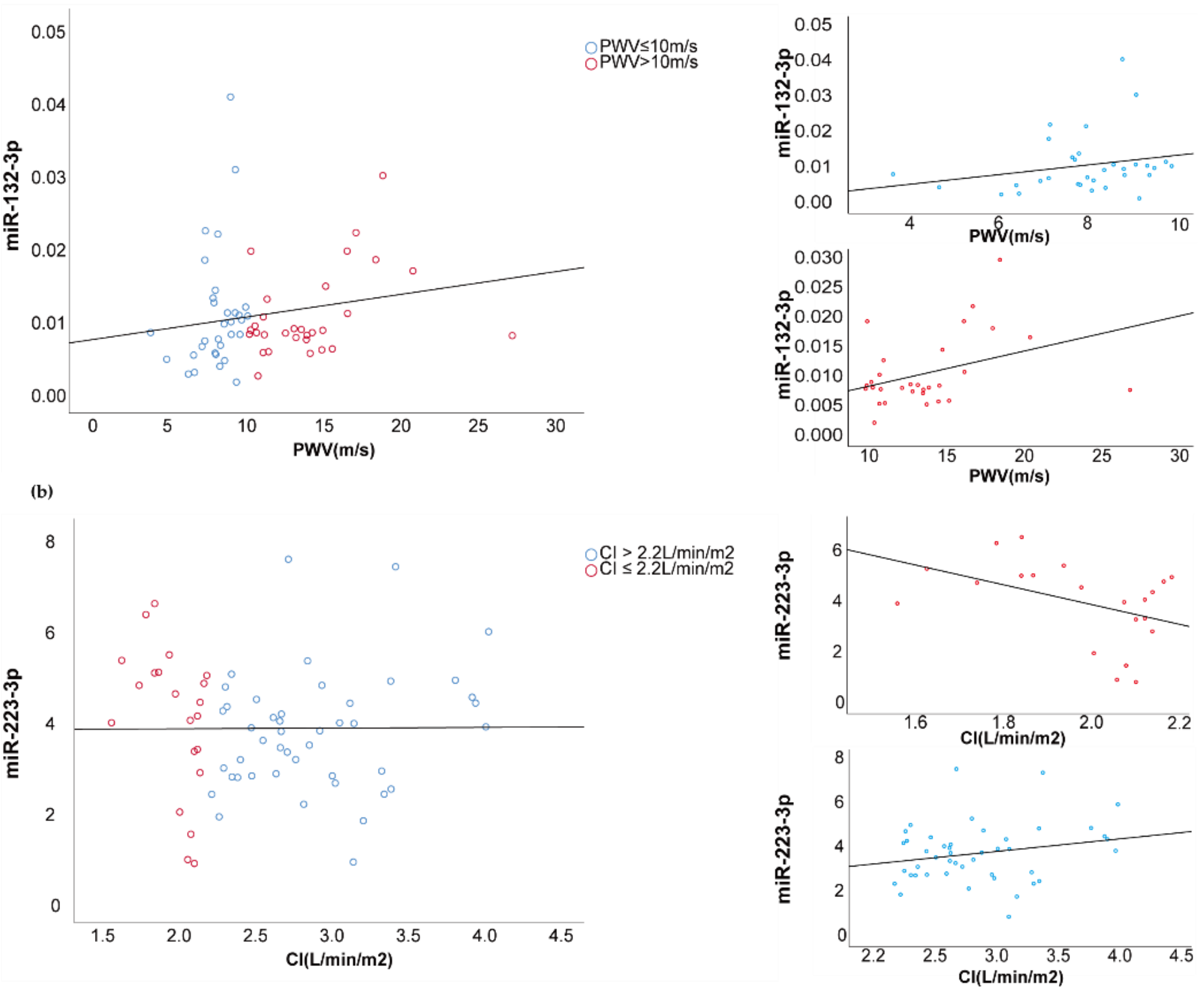

Supplementary Figure S1. Blue dots represent better cardiovascular condition patients whose PWV ≤ 10 m/s or CI > 2.2 L/min/m², and red dots represent worse cardiac condition patients whose PWV > 10 m/s or CI ≤2.2 L/min/m². (a) PWV was significantly correlated with miR-132 (R = 0.371, P = 0.043) when PWV > 10 m/s, while not existed (R = 0.230, P = 0.205) when PWV ≤ 10 m/s. (b) CI was negatively correlated with miR-223 (R= -0.440, P = 0.046) within the group with a CI ≤2.2 L/min/m² . There is no significant correlation between CI and miR-223 (R = 0.220, P = 0.147) when CI > 2.2 L/min/m².

Supplementary Table S1. Correlations between vascular injury markers and cardiovascular function parameters within dichotomized groups.

	**Pulse wave velocity ≤ 10 m/s n = 35**		**Pulse wave velocity > 10 m/s n = 32**	
	R	P value	R	P value
ADMA	0.062	0.725	-0.075	0.681
ANG2	0.016	0.931	0.583	0.001**
miR-126	-0.023	0.893	0.019	0.916
miR-132	0.230	0.205	0.371	0.043*
miR-192	0.016	0.931	0.224	0.233
miR-223	-0.233	0.178	-0.225	0.215
miR-27	-0.232	0.187	-0.379	0.033*
miR-29	0.085	0.626	0.102	0.578
miR-326	0.163	0.366	0.164	0.425
	**Ejection fraction ≥ 50 % n = 54**		**Ejection fraction < 50 % n = 13**	
	R	P value	R	P value
ADMA	0.045	0.744	0.199	0.515
ANG2	-0.229	0.113	-0.046	0.880
miR-126	-0.104	0.453	0.211	0.488
miR-132	0.004	0.978	-0.022	0.945
miR-192	-0.027	0.856	0.209	0.494
miR-223	-0.119	0.390	0.192	0.529
miR-27	-0.006	0.967	0.063	0.846
miR-29	0.176	0.203	0.026	0.932
miR-326	-0.110	0.452	0.022	0.953
	**Cardiac index > 2.2 L/min/m² n = 45**		**Cardiac index ≤ 2.2 L/min/m² n = 21**	
	R	P value	R	P value
ADMA	0.064	0.678	-0.019	0.934
ANG2	0.064	0.690	0.215	0.362
miR-126	0.069	0.654	-0.218	0.342
miR-132	-0.154	0.337	0.405	0.076
miR-192	-0.093	0.561	0.094	0.702
miR-223	0.220	0.147	-0.440	0.046*
miR-27	0.001	0.993	-0.244	0.301
miR-29	0.032	0.834	0.290	0.202
miR-326	-0.140	0.396	0.078	0.752

Bivariate correlation models were used for the correlation p-values. Numbers of patients in each group is showed by n. * $p < 0.05$, ** $p < 0.01$
